# The Potential Impact of Heparanase Activity and Endothelial Damage in COVID-19 Disease

**DOI:** 10.3390/jcm11185261

**Published:** 2022-09-06

**Authors:** Elisabeth Zechendorf, Katharina Schröder, Lara Stiehler, Nadine Frank, Christian Beckers, Sandra Kraemer, Michael Dreher, Alexander Kersten, Christoph Thiemermann, Gernot Marx, Tim-Philipp Simon, Lukas Martin

**Affiliations:** 1 Department of Intensive and Intermediate Care, University Hospital RWTH Aachen, 52074 Aachen, Germany; 2 Department of Pneumology and Intensive Care Medicine, University Hospital RWTH Aachen, 52074 Aachen, Germany; 3 William Harvey Research Institute, Barts and The London School of Medicine and Dentistry, Queen Mary University of London, London EC1M 6BQ, UK

**Keywords:** COVID-19, ARDS, endothelial dysfunction, heparanase, heparan sulfate, biomarker

## Abstract

SARS-CoV-2 was first detected in 2019 in Wuhan, China. It has been found to be the most pathogenic virus among coronaviruses and is associated with endothelial damage resulting in respiratory failure. Determine whether heparanase and heparan sulfate fragments, biomarkers of endothelial function, can assist in the risk stratification and clinical management of critically ill COVID-19 patients admitted to the intensive care unit. We investigated 53 critically ill patients with severe COVID-19 admitted between March and April 2020 to the University Hospital RWTH Aachen. Heparanase activity and serum levels of both heparanase and heparan sulfate were measured on day one (day of diagnosis) and day three in patients with COVID-19. The patients were classified into four groups according to the severity of ARDS. When compared to baseline data (day one), heparanase activity increased and the heparan sulfate serum levels decreased with increasing severity of ARDS. The heparanase activity significantly correlated with the lactate concentration on day one (*r* = 0.34, *p* = 0.024) and on day three (*r* = 0.43, *p* = 0.006). Heparanase activity and heparan sulfate levels correlate with COVID-19 disease severity and outcome. Both biomarkers might be helpful in predicting clinical course and outcomes in COVID-19 patients.

## 1. Introduction

Coronaviruses have been known since 1960. A total of seven different coronaviruses have been described, including the coronavirus SARS-CoV-2 [1]. Most coronaviruses are associated with a moderate clinical course with mild respiratory diseases. The coronaviruses SARS-CoV and MERS-CoV are associated with a more severe disease course and higher mortality. SARS-CoV was first identified in 2003. A total of 8422 cases were detected, and 916 fatalities were described [2]. Having a mortality rate of 34.4%, the MERS-CoV virus turned out to be one of the most dangerous viruses of the group of coronaviruses. From 2012 until today, 2500 cases have been confirmed [3]. SARS-CoV-2 was first detected in 2019 in Wuhan, China. It has been found to be the most pathogenic virus among the coronaviruses [4]. SARS-CoV-2 is associated with a variation in respiratory syndromes ranging from mild airway symptoms to life-threatening viral pneumonia. One-third of all lung cells are endothelial cells, which are known to be associated with the severity of lung damage in patients [5]. The endothelial glycocalyx coats the surface layer of endothelial cells. The endothelial glycocalyx interacts with the blood and thereby regulates microcirculatory flow [6]. The endothelium has a key role in the innate immune response in a wide array of critical care conditions [5]. Endothelial cells are also involved in maintaining barrier function and preventing inflammation by limiting their interactions with immune cells and platelets [6]. Endothelial dysfunction is a key driver in the pathogenesis of the organ dysfunction during viral infections [7]. Angiotensin-converting enzyme 2 (ACE2) is expressed by endothelial cells and has been found in a variety of arterial and venous endothelial cells [8]. SARS-CoV-2 enters target cells and initiates infection by binding to ACE2 on the cell membrane of host cells [8,9]. SARS-CoV-2 has been detected in endothelial cells of many organs. For instance, Varga et al. described that patients with COVID-19 exhibit endothelial cell injury in blood vessels of the kidney, liver, heart, and lung [10]. Monteil and colleagues reported that SARS-CoV-2 directly infects engineered human blood vessels in vitro [11]. Most notably, endothelial cell injury has also been confirmed by transmission electron microscopy in blood vessels obtained during autopsy of patients who died from COVID-19 [6].

The endothelial glycocalyx is composed of different glycoproteins, proteoglycans, and glycosaminoglycans. The heparan sulfate proteoglycan is one of these proteoglycans and represents approximately 50–90% of the total amount of proteoglycans present in the glycocalyx [12]. As a consequence of infection, sheddases, such as heparanase, are activated. Heparanase specifically cleaves heparan sulfate fragments from the proteoglycan, resulting in a loss of integrity and, thus, in endothelial dysfunction. Therefore, it is hypothesized that increased heparanase activity may be one of the driving forces in severe COVID-19 manifestations. Indeed, Buijsers and colleagues demonstrated that heparanase activity and heparan sulfate levels are significantly increased in the plasma of COVID-19 patients, which is related to the severity of the disease [13]. Furthermore, another study showed a significantly higher heparanase activity and increased levels of heparan sulfate in plasma of COVID-19 patients [14].

There is strong evidence that endothelial dysfunction plays a critical role in the pathophysiology and clinical course of acute respiratory distress syndrome (ARDS) [15]. However, the role of heparanase activity and heparanase and heparan sulfate serum levels in this context is unclear. Therefore, the aim of this study was to investigate the relationship between heparanase activity and heparan sulfate fragment formation and outcome in COVID-19 patients and to determine the prognostic value of these potential new biomarkers in ARDS.

## 2. Materials and Methods

### 2.1. Study Design

After approval by the Ethics Committee of the University Hospital RWTH Aachen (EK 100/20), serum samples were collected between March and April 2020. All patients or their legal representatives provided written informed consent. After excluding patients younger than 18 years of age who were pregnant or under palliative care, 53 patients with positive SARS-CoV-2 PCR results and intensive care admission were included in this study. Identification of infection was carried out using real-time reverse transcription PCR (RT-PCR). Treatment of patients followed the standards of care in our intensive care unit (ICU), including mechanical ventilation, veno-venous extracorporeal membrane oxygenation (ECMO) and renal replacement therapy (RRT) if needed. The decision to use veno-venous ECMO therapy was based on the recently published Extracorporeal Life Support Organization (ELSO) consensus guidelines [16]. All parameters, including demographics, vital signs, laboratory values, blood gas analyses, and organ support, were extracted from the patient data management system (Intellispace Critical Care and Anesthesia (ICCA) system, Philips, The Netherlands).

### 2.2. Serum Sampling

Serum samples were collected at two different time points after patients were enrolled in the study following a positive SARS-CoV-2 PCR result: on the day of the positive SARS-CoV-2 PCR result (day 1) and two days after COVID-19 diagnosis (day 3). Serum samples were centrifuged at 3000 rpm for 10 min at room temperature after a 10-min clotting period. Samples were stored at −80 °C until heparanase activity and heparanase as well as heparan sulfate serum levels were measured.

### 2.3. Heparanase Activity Assay

Heparanase activity was detected using a commercial heparanase assay kit (Cat. #: Ra001-BE-K, Amsbio, Abingdon, UK). The measurements were performed according to the manufacturer’s instructions. After rehydration of the microwell plate, 100 µL of the positive control enzyme was added to two wells. For sample analysis, 50 µL of the reaction buffer and 50 µL of the samples were added to each well. All measurements were performed in duplicate. For the negative control, the reaction buffer alone was used. The microwell plate was incubated at 37 °C for one hour on a plate shaker and washed afterwards. Next, 100 µL Strep-HRP was added to each well, and the plate was incubated at 37 °C for 50 min while shaking. The microwell plate was washed again followed by incubation at room temperature with 100 µL peroxidase substrates. The absorbance was measured at intervals of 1 to 2 min at 650 nm using a microplate reader (Infinity 200, Tecan, Männedorf, Switzerland). The reaction was stopped by adding 100 µL of 0.12 M HCl to each well as soon as an optical density of 0.6–0.7 was reached. The optical density was determined at 450 nm.

### 2.4. Human Enzyme-Linked Immunosorbent Assay

Heparanase and heparan sulfate serum levels were detected using commercial ELISA kits (Cat. #: E01H0100 and Cat. #: E01H1352, amsbio). For analysis, 100 µL of the standard and samples were added to the precoated wells. As negative control, 100 µL of PBS was used. All measurements were performed in duplicate. An additional 50 µL conjugate was added to each well except for the negative control. After mixing, the microplate was incubated for 1 h at 37 °C. The microplate was washed five times with wash solution, and 50 µL substrate was added. The reaction was stopped with 50 µL of the stop solution after 10–15 min of incubation at room temperature. The optical density was determined at 450 nm using a microplate reader (Infinity 200, Tecan, Männedorf, Switzerland). Sample values were interpolated from the 4PL regression standard curve generated by GraphPad Prism 7 (GraphPad by Dotmatics, San Diego, CA, USA).

### 2.5. Statistics

Values are expressed as medians and interquartile ranges (IQRs) or counts and percentages, as appropriate. Group comparisons of continuous variables were performed using the Kruskal–Wallis test. Post hoc tests were computed according to Siegel and Castellan 1988 [17]. Categorical data were compared using Pearson’s chi-squared test for count data. Biomarker data are typically log-normally distributed and were, therefore, log-transformed for statistical analysis. The untransformed data are presented in a boxplot to illustrate the association of continuous variables with categorical variables. The lines inside the boxes represent the median and the box ranges from the lower quartile (Q1) to the upper quartile (Q3). The circles outside the box are values that represent outliers. Any data observation that was more than 1.5 IQR below the first quartile or above the third quartile is considered an outlier. The smallest/largest value that was not an outlier was indicated by a vertical marker or “whisker”. The whisker is connected to the box by a horizontal line. Cox proportional hazards regression was used to analyze the effect of the (log-transformed) biomarker on survival in univariable analyses. Survival curves plotted by the Kaplan–Meier method were used for illustrative purposes. Cox models are slightly positively affected by using log-transformed data. All other results (Kruskal–Wallis test, Spearman r, Kaplan–Meier plots) are not affected by the log transformation since they are rank-based. All statistical tests were 2-tailed, and a two-sided p-value of 0.05 was considered significant. Statistical analyses were performed using R version 3.4.3 (http://www.r-project.org (accessed on 27 July 2020), library rms, Hmisc, ROCR, Vienna, Austria) and Statistical Package for the Social Sciences (SPSS) version 22.0 (SPSS Inc., Chicago, IL, USA).

## 3. Results

In this study, 53 ICU patients with a confirmed SARS-CoV-2 infection were included. The patients were classified into four groups according to the severity of ARDS. Three (5.7%) patients did not develop ARDS, 12 (22.6%) patients developed mild ARDS, 13 (24.5%) developed moderate ARDS, and most patients 25 (47.2%) developed severe ARDS. Patients without ARDS were in the ICU for a mean of 6 days, patients with mild ARDS for 7.5 days, patients with moderate ARDS for 19.5 days and patients with severe ARDS for 17.5 days. Of these, one (7.7%) patient with moderate ARDS and 7 (28.0%) patients with severe ARDS died in the ICU. Thus, the overall mortality of COVID-19 patients admitted to ICU was 15%. Detailed patient characteristics are shown in Table 1.

**Figure 1:**
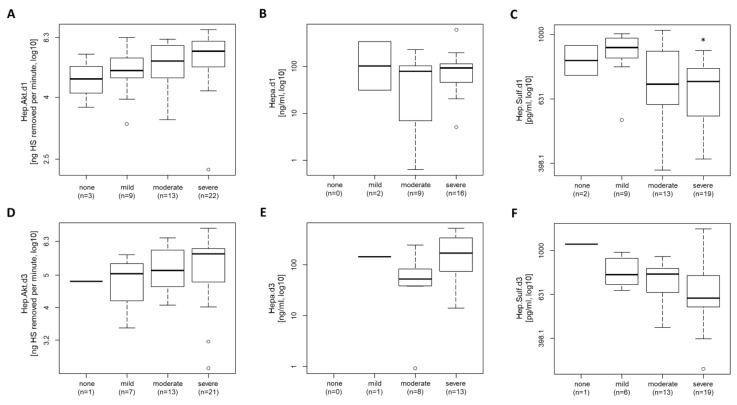
Heparanase activity, heparanase level, and heparan sulfate level in COVID-19 patients in relation to the severity of ARDS. Boxplot of heparanase activity on (**A**) day 1 and (**D**) 3 and heparanase level on (**B**) day 1 and (**E**) 3, as well as the heparanase sulfate level on (**C**) day 1 and (**F**) 3 of COVID-19 patients in relation to the severity of ARDS (none, mild, moderate, or severe ARDS) are shown. The untransformed data are presented as a boxplot. The lines inside the boxes represent the median, and the box is defined by the range of Q1 and Q3. Circles outside the box are values represent outliers. For statistical analysis, Kruskal–Wallis analysis was used with a significance level of *p* < 0.05, with the post hoc test indicating which groups showed a statistical difference by TRUE (significant) or FALSE (not significant). * indicates a significant difference between severe and mild ARDS. ARDS: acute respiratory distress syndrome; Hep.Akt.: heparanase activity; Hepa.: heparanase; Hep.Sulf.: heparan sulfate.

On day one (the day of inclusion into the study), we measured an increased heparanase activity in patients with a severe ARDS, but no significant difference was detected between patients with different severity of ARDS (Figure 1A, *p* = 0.144). There was no difference in heparanase serum levels detected between patients with different severities of ARDS (Figure 1B, *p* = 0.640). When compared to patients that had not developed ARDS, patients with mild ARDS showed a small, but significant increase in the serum levels of heparan sulfate, while the heparan sulfate serum levels were lower in patients with moderate or severe ARDS (compared to patients with either mild or no ARDS; Figure 1C, *p* = 0.022). Three days after ICU admission, mean heparanase serum levels in patients with moderate ARDS showed a tendency toward slightly lower levels compared with patients with severe ARDS (Figure 1E). Also, no significant differences in serum heparanase levels were observed on the third day between patients with different severity of ARDS (Figure 1E, *p* = 0.140). Heparanase activity and heparan sulfate serum levels showed similar trends as on day 1 (Figure 1D,F, *p* = 0.470 and *p* = 0.129).

The heparanase activity significantly correlated (positively) with the lactate concentration on day one (*r* = 0.34, *p* = 0.024, CI: 0.07, 0.58) and on day three (*r* = 0.43, *p* = 0.006, CI: 0.01, 0.47). Additionally, a significant positive correlation could be detected with PCT on both days (*r* = 0.38, *p* = 0.011, CI: 0.09, 0.65 and *r* = 0.36, *p* = 0.023, CI: 0.04, 0.60). Furthermore, heparanase activity significantly correlated positively with CRP on day one (*r* = 0.34, *p* = 0.020, CI: −0.03, 0.59) and IL-6 on day three (*r* = 0.36, *p* = 0.032, CI: −0.12, 0.54). Interestingly, a slightly positive non-significant correlation of heparanase serum levels and a negative significant correlation of heparan sulfate serum levels with PCT were measured on day three (*r* = 0.40, *p* = 0.065, CI: −0.04,0.72; *r* = −0.35, *p* = 0.034, CI: −0.58, −0.02). The heparan sulfate serum level also correlated negatively with the SOFA score on day one (*r* = −0.35, *p* = 0.028, CI: −0.57, −0.06; Table 2).

Next, we analyzed the potential of heparan sulfate and heparanase serum levels as well as heparanase activity to predict 28-day mortality. None of the biomarkers showed a linear association with mortality (all *p* > 0.20), nor a consistent non-linear association between day 1 and day 3. For illustration, the biomarker levels were divided into 4 quartiles and analyzed by using a Kaplan–Meier plot with respect to 28-day mortality. Patients with higher heparanase levels [113–600] on day one had a 100% survival rate. In contrast, patients with heparanase levels between 86.3–113.0 ng/mL on day one had a higher probability of dying within 28 days (below 60% survival).

**Figure 2:**
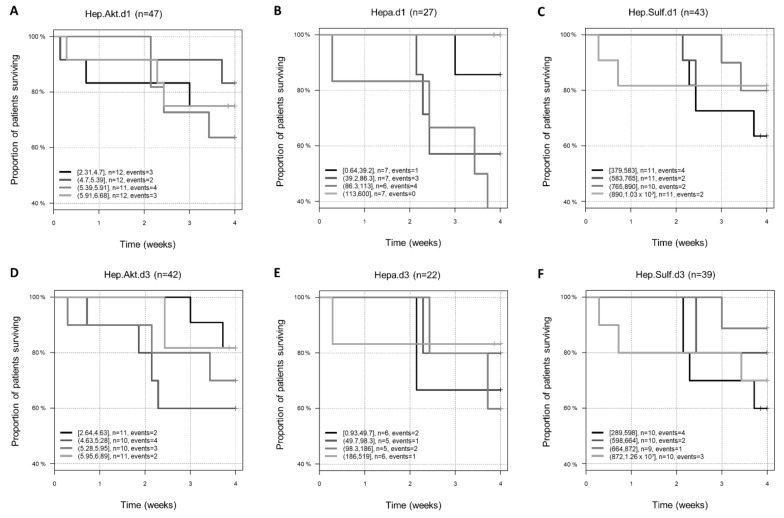
The relationship between heparan sulfate and heparanase serum levels as well as heparanase activity and the 28-day mortality. Presented are Kaplan–Meier plots for 28-day mortality for heparanase activity on (**A**) day 1 and (**D**) day 3 and heparanase level on (**B**) day 1 and (**E**) day 3 as well as the heparanase sulfate level on (**C**) day 1 and (**F**) day 3 of COVID-19 patients. Curves are plotted by quartiles. Hep.Akt.: heparanase activity; Hepa: heparanase; Hep.Sulf.: heparan sulfate.

Patients presenting heparan sulfate levels below 583 ng/mL on day one and below 598 ng/mL on day three showed the worst outcome. Patients with heparanase activity in the range of the third quarter on days one [5.39; 5.91] and three [5.28; 5.95] also showed a worse outcome. Patients with heparanase activity in the range of the second quarter [4.63; 5.28] showed an even worse outcome, with only 60% of patients surviving (Figure 2).

## 4. Discussion

The new coronavirus represents a major challenge, especially for intensive care units, because SARS-CoV-2 is one of the most pathogenic coronaviruses, and a high proportion of patients suffer a severe course of disease [18]. Endothelial dysfunction plays a crucial role in ARDS and consequently in the pathophysiology of COVID-19. In a previous study, Buijsers et al. postulated an association between elevated heparanase activity and heparan sulfate plasma levels and the need for intensive care unit admission [13]. In fact, our work confirms and extends this data by showing a clear potential of heparanase activity and heparan sulfate plasma levels as promising biomarkers for the prediction of disease severity and outcome of COVID-19 patients admitted to the ICU.

Consequently, in this work, the role of heparanase and heparan sulfate in COVID-19 patients in relation to the severity of ARDS was investigated. Indeed, in patients with severe ARDS, an increase in the activity of heparanase on day 1 (5.7 [5.1–6.1]) and day 3 (5.8 [4.8–6.0]) was observed when compared to patients with moderate (day 1: 5.3 [4.6–5.9], day 3: 5.2 [4.6–5.9]) and mild ARDS (day 1: 4.9 [4.6–5.4]; day 3: 5.0 [4.3–5.4]) (Figure 1). Unlike with heparanase activity, we did not observe an increase in heparanase levels with an increase in ARDS severity. Along with increasing severity of ARDS, there is an increased expression of proinflammatory cytokines, such as IL-6. Heparanase is activated by proinflammatory cytokines [19]; thus, the increased activity with respect to severe ARDS might be associated with the increased expression of proinflammatory cytokines, rather than with an increase in heparanase levels. On day 3, a correlation of heparanase activity and IL-6 concentration was detected (Table 2). This result confirms the assumption that IL-6 is involved in heparanase activation. However, no elevated heparan sulfate serum levels could be measured in COVID-19 patients with severe ARDS compared to those with moderate or mild ARDS. In line, Buijsers et al. showed that a reduction in heparanase activity due to the prophylactic administration of low molecular weight heparins (LMWH) was not associated with a reduction in heparan sulfate or IL-6 plasma levels [13].

In addition to the fragmentation of heparan sulfate (and its fragments), heparanase is involved in the cleavage of heparan sulfate-bound cytokines and growth factors such as angiopoietin-2 (Ang-2). In various studies, a significantly higher Ang-2 concentration was measured in the serum of septic patients compared to healthy subjects [20,21]. Furthermore, increased heparan sulfate and heparanase levels in the serum of patients and mice with sepsis [22,23,24] suggests an association between heparanase activity and increased expression of Ang-2. Lukasz and colleagues showed that the Ang-2-mediated breakdown of endothelial glycocalyx thickness depends on heparanase [25]. Furthermore, increased Ang-2 levels were detected in COVID-19 patients [26]. Drost et al. showed that HPSE activity was inversely correlated with HS concentration in healthy volunteers and COVID-19 patients [14]. This would also be in line with our results, as we measured that heparan sulfate levels tended to decrease with increasing severity of ARDS in COVID-19 patients.

## 5. Limitation/Conclusions

As our investigation was limited to a small patient cohort with a variation in the number of measurements of some variables, further investigations in a larger cohort should be carried out to confirm our data. Moreover, our measurements were limited to heparanase activity and heparanase as well as heparan sulfate serum levels, and the analysis of other biomarkers associated with activation of the heparanase system, such as syndecane-1 concentrations in serum, would strengthen the findings reported here.

Due to the novel SARS-CoV-2 variants, COVID-19 still plays a crucial role today. Therefore, it is still important to identify new biomarkers that indicate the severity of COVID-19 and the severity of COVID-19-associated ARDS. In conclusion, in this study, we showed that heparanase activity increased in patients with severe ARDS on both days in COVID-19 patients. Furthermore, heparanase activity and heparan sulfate levels correlate with COVID-19 disease severity and outcome. Based on these results, there is now good evidence that heparanase activity may play a role in endothelial dysfunction and ARDS associated with COVID-19 and may be a potential biomarker to predict outcome in COVID-19 patients in the ICU. This may also be relevant to newer variants of SARS-CoV-2.

## Figures and Tables

**Table 1:** Patient characteristics separated by severity of ARDS.

Variable	None (*n* = 3)	Mild (*n* = 12)	Moderate (*n* = 13)	Severe (*n* = 25)	*p*-Value
***n*** **(%)**	3 (5.7)	12 (22.6)	13 (24.5)	25 (47.2)	
**Age (years)**	53 [49–65]	61 [59–64]	62 [54–67]	66 [58–72]	0.767
**Sex male, *n* (%)**	3 (100.0)	10 (83.3)	6 (46.2)	21 (84.0)	0.039
**Body mass index (kg/m^2^)**	24.9 [24.7–28.2]	29.2 [26.3–34.9]	30.5 [26.7–35.2]	29.3 [24.7–31.3]	0.759
**Temperature, max (°C)**	38.1 [37.9–38.9]	38.5 [38.2–38.7]	38.15 [37.7–38.8]	38.1 [37.4–38.6]	0.229
**Heart rate (bpm)**	89.0 [84.5–105.5]	108.0 [99.0–112.0]	108.0 [87.0–123.5]	112.0 [102.0–124.0]	0.519
**Respiratory rate (bpm)**	24.0 [23.5–25.0]	25.0 [24.0–27.0]	24.0 [23.0–26.0]	24.0 [20.0–27.5]	0.988
**SOFA score at day of enrollment (points)**	8.5 [7.6–9.3]	8.0 [5.3–9.8]	10.0 [8.0–12.3]	11.0 [9.0–12.0]	0.034
**Blood gas analysis (at day of enrollment)**					
**Arterial pH**	7.5 [7.4–7.5]	7.4 [7.4–7.5]	7.4 [7.3–7.4]	7.4 [7.3–7.4]	0.040
**pCO_2_ (mmHg)**	48.0 [42.1–52.1]	36.3 [33.0–38.9]	47.5 [42.7–54.0]	49.8 [42.1–61.1]	0.025
**pO_2_ (mmHg)**	83.0 [77.0–85.5]	97.0 [80.0–107.0]	71.0 [68.0–74.8]	82.0 [71.0–93.0]	0.188
**SpO_2_ (%)**	94.0 [92.5–94.5]	98.0 [95.8–98.3]	96.0 [92.8–99.0]	95.0 [92.0–97.0]	0.053
**Horowitz index (mmHg/%)**	93.0 [68.5–256.0]	218.0 [179.8–229.3]	120.5 [105.8–135.3]	95.0 [77.0–125.0]	0.001
**Biomarker (at day of enrollment, unless stated differently)**					
**Lactate (mmol/L)**	0.7 [0.7–1.0]	0.7 [0.4–0.9]	1.2 [0.8–1.6]	1.4 [1.0–1.6]	0.030
**IL-6 (pg/mL)**	86.8 [86.8–86.8]	60.3 [37.9–93.3]	496.3 [158.7–623.7]	263.3 [105.6–708.9]	0.018
**PCT (ng/mL)**	0.1 [0.1–0.1]	0,3 [0.1–0.4]	0,.4 [0.2–0.7]	1.5 [0.7–5.7]	<0.001
**CRP (mg/L)**	103.2 [63.7–142.6]	115.9 [92.7–144,0]	281.6 [200.8–313.4]	277.4 [186.8–337.8]	0.003
**Leukocytes (10^3^/mm^3^) =/nl**	10.1 [9.2–11.7]	6.9 [6.3–10.8]	9.2 [7.1–10.6]	8.9 [7.8–14.1]	0.362
**Platelets (10/µL)**	202.0 [178.5–292.0]	200.0 [168.0–284.0]	276.5 [197.8–338.8]	247.0 [185.0–307.0]	0.551
**Creatinine (mg/dL)**	0.7 [0.6–0.7]	0.9 [0.7–1.4]	0.9 [0.6–1.2]	1.8 [1.0–2.7]	0.003
**Comorbidities**					
**Arterial hypertension, *n* (%)**	1 (33.3)	5 (41.7)	9 (69.2)	12 (48.0)	0.455
**Diabetes mellitus, *n* (%)**	0 (0.0)	1 (8.3)	3 (23.1)	9 (36.0)	0.215
**Adipositas, *n* (%)**	0 (0.0)	2 (16.7)	4 (30.8)	6 (24.0)	0.651
**Hyper-/Dyslipidemia, *n* (%)**	0 (0.0)	1 (8.3)	3 (23.1)	3 (12.0)	0.606
**Ischemic heart disease, *n* (%)**	0 (0.0)	2 (16.7)	4 (30.8)	4 (16.0)	0.557
**Embolism/Thrombosis, *n* (%)**	1 (33.3)	1 (8.3)	3 (23.1)	1 (4.0)	0.197
**Cardiac arrhythmia, *n* (%)**	0 (0.0)	1 (8.3)	0 (0.0)	5 (20.0)	0.259
**Peripheral arterial occlusive disease, *n* (%)**	0 (0.0)	1 (8.3)	1 (7.7)	0 (0.0)	0.506
**Cerebral vascular disease,** ***n* (%)**	0 (0.0)	2 (16.7)	0 (0.0)	3 (12.0)	0.459
**COPD, *n* (%)**	1 (33.3)	2 (16.7)	0 (0.0)	2 (8.0)	0.525
**bronchial asthma, *n* (%)**	0 (0.0)	0 (0.0)	3 (23.1)	1 (4.0)	0.104
**Other lung diseases, *n* (%)**	1 (33.3)	1 (8.3)	0 (0.0)	0 (0.0)	0.025
**Chronic kidney disease, *n* (%)**	0 (0.0)	2 (16.7)	3 (23.1)	3 (12.0)	0.708
**Tumor disease, *n* (%)**	0 (0.0)	3 (25.0)	1 (7.7)	0 (0.0)	0.057
**Smoker, *n* (%)**	0 (0.0)	1 (8.3)	2 (15.4)	0 (0.0)	0.247
**Ex-Smoker, *n* (%)**	0 (0.0)	1 (8.3)	2 (15.4)	1 (4.0)	0.604
**Medication at admission**					
**Anticoagulation, *n* (%)**	1 (33.3)	2 (16.7)	3 (23.1)	9 (36.0)	0.627
**Antiplatelet, *n* (%)**	0 (0.0)	4 (33.3)	6 (46.2)	5 (20.0)	0.238
**Antihypertensives, *n* (%)**	1 (33.3)	8 (66.7)	10 (76.9)	13 (52.0)	0.343
**Immunosupressants, *n* (%)**	1 (33.3)	2 (16.7)	4 (30.8)	2 (8.0)	0.289
**Analgesics, *n* (%)**	1 (33.3)	4 (33.3)	1 (7.7)	2 (8.0)	0.143
**Treatment on ICU (first 14 days, unless stated differently)**					
**ICU length of stay (days)**	6.0 [4.0–9.5]	7.5 [3.0–10.5]	19.5 [16.5–22.8]	17.5 [15.0–20.8]	0.004
**Highest dose of norepinephrine during the first 7 days (µg/kg/min)**	0.1 [0.0–0.1]	0.0 [0.0–0.0]	0.1 [0.1–0.2]	0.1 [0.1–0.21]	0.002
**Duration of ventilation (hours)**	103.0 [51.5–147.0]	0.0 [0.0–218.8]	330.0 [271.0–341.0]	331.0 [246.0–349.0]	<0.001
**Duration of RRT (days)**	0 [0–0]	0 [0–0]	0.0 [0–8]	12 [1–14]	0.001
**ECMO**					0.471
**never, *n* (%)**	3 (100.0)	12 (100.0)	11 (84.6)	18 (72.0)	
**at admission, *n* (%)**	0 (0.0)	0 (0.0)	1 (7.7)	5 (20.0)	
**later, *n* (%)**	0 (0.0)	0 (0.0)	1 (7.7)	2 (8.0)	
**Ventilation**					
**Intubation, *n* (%)**	1 (33.3)	5 (41.7)	13 (100.0)	25 (100.0)	
**Never**	2 (66.7)	7 (58.3)	0 (0.0)	0 (0.0)	
**at admission**	1 (33.3)	3 (25.0)	12 (92.3)	22 (88.0)	
**Later**	0 (0.0)	2 (16.7)	1 (7.7)	3 (12.0)	
**External intubation, *n* (%)**	1 (33.3)	0 (0.0)	4 (30.8)	19 (76.0)	
**Mechanical ventilation, *n* (%)**	2 (66.7)	5 (41.7)	13 (100.0)	25 (100.0)	
**FiO_2_ (%)**	87.5 [81.3–93.8]	40.0 [37.5–41.3]	60.0 [55.0–71.3]	70.0 [60.0–86.0]	0.004
**PEEP (cmH_2_O)**	15 [15.0–15.0]	8 [7.0–9.0]	13 [12.0–15.0]	15 [13.5–16.0]	0.002
**Tidal volume (mL)**	600.5 [560.8–640.3]	674.0 [564.0–810.0]	620.0 [521.8–737.8]	503.5 [435.0–600.0]	0.045
**NIV, *n* (%)**	2 (66.7)	1 (8.3)	3 (23.1)	2 (8.0)	0.041
**Outcome**					
**Death at 28 days, *n* (%)**	1 (33.3)	0 (0.0)	1 (7.7)	11 (44.0)	0.011
**Disposition on day 28**					0.001
**discharged, *n* (%)**	2 (66.7)	12 (100.0)	11 (84.6)	7 (28.0)	
**on ICU post day 28, ** ***n* (%)**	0 (0.0)	0 (0.0)	1 (7.7)	7 (28.0)	
**Death at 28 days, *n* (%)**	1 (33.3)	0 (0.0)	1 (7.7)	11 (44.0)	

Variables are given as median [interquartile range] or number (%). Kruskal–Wallis analysis with a significance level of *p* < 0.05 was used for statistical analysis. ARDS, acute respiratory distress syndrome; COPD, chronic obstructive pulmonary disease; CRP, C-reactive protein; ECMO, extracorporeal membrane oxygenation; FiO_2_, fraction of inspired oxygen; ICU, intensive care unit; IL-6, interleukin-6; pCO_2_, partial pressure of carbon dioxide; PCT, procalcitonin; pO_2_, partial pressure of oxygen; RRT, renal replacement therapy; SpO_2_, peripheral capillary oxygen saturation; SOFA, sequential organ failure assessment.

**Table 2:** Significant correlations between biomarkers and clinical features.

Variable	Spearman *r*	*p*-Value	CI
**Heparanase level d1**			
**Age**	−0.34	0.0805	−0.69, 0.06
**SpO_2_**	−0.44	0.0297	−0.77, −0.07
**Leucocytes**	0.50	0.0103	0.07, 0.79
**Heparan sulfate level d1**			
**BMI**	0.31	0.0467	−0.06, 0.57
**Respiratory rate**	−0.42	0.0201	−0.63, −0.11
**SOFA-Score**	−0.35	0.0281	−0.57, −0.06
**Norepinephrine**	−0.31	0.0397	−0.56, 0.02
**Heparanase activity d1**			
**Lactate**	0.34	0.0241	0.07, 0.58
**PCT**	0.38	0.0113	0.09, 0.65
**CRP**	0.34	0.0200	−0.03, 0.59
**Heparanase level d3**			
**Duration of ventilation**	−0.47	0.0273	−0.82, 0.03
**Temperature max**	−0.51	0.0158	−0.76, −0.13
**Heparan sulfate level d3**			
**PCT**	−0.35	0.0341	−0.58, −0.02
**Leucocytes**	−0.44	0.0055	−0.61, −0.17
**Heparanase activity d3**			
**Lactate**	0.43	0.0055	0.01, 0.47
**IL-6**	0.36	0.0322	−0.12, 0.54
**PCT**	0.36	0.0226	0.04, 0.60

Shown are significant correlations of heparanase activity, heparanase serum level, and heparan sulfate serum level at days 1 and 3 with various clinical parameters, and Spearman rank correlation coefficient r with 95% confidence interval CI (bootstrap CI, B = 200). BMI, body mass index; CRP, C-reactive protein; IL-6, interleukin-6; PCT, procalcitonin; SpO_2_, peripheral capillary oxygen saturation; SOFA, sequential organ failure assessment.

## Data Availability

Not applicable.
